# The *ACR11 *encodes a novel type of chloroplastic ACT domain repeat protein that is coordinately expressed with *GLN2 *in *Arabidopsis*

**DOI:** 10.1186/1471-2229-11-118

**Published:** 2011-08-24

**Authors:** Tzu-Ying Sung, Tsui-Yun Chung, Chih-Ping Hsu, Ming-Hsiun Hsieh

**Affiliations:** 1 Institute of Plant and Microbial Biology, Academia Sinica, Taipei 11529, Taiwan

## Abstract

**Background:**

The ACT domain, named after bacterial aspartate kinase, chorismate mutase and TyrA (prephenate dehydrogenase), is a regulatory domain that serves as an amino acid-binding site in feedback-regulated amino acid metabolic enzymes. We have previously identified a novel type of ACT domain-containing protein family, the ACT domain repeat (ACR) protein family, in *Arabidopsis*. Members of the ACR family, ACR1 to ACR8, contain four copies of the ACT domain that extend throughout the entire polypeptide. Here, we describe the identification of four novel ACT domain-containing proteins, namely ACR9 to ACR12, in *Arabidopsis*. The ACR9 and ACR10 proteins contain three copies of the ACT domain, whereas the ACR11 and ACR12 proteins have a putative transit peptide followed by two copies of the ACT domain. The functions of these plant ACR proteins are largely unknown.

**Results:**

The ACR11 and ACR12 proteins are predicted to target to chloroplasts. We used protoplast transient expression assay to demonstrate that the *Arabidopsis *ACR11- and ACR12-green fluorescent fusion proteins are localized to the chloroplast. Analysis of an *ACR11 *promoter-β-glucuronidase (GUS) fusion in transgenic *Arabidopsis *revealed that the GUS activity was mainly detected in mature leaves and sepals. Interestingly, coexpression analysis revealed that the *GLN2*, which encodes a chloroplastic glutamine synthetase, has the highest mutual rank in the coexpressed gene network connected to *ACR11*. We used RNA gel blot analysis to confirm that the expression pattern of *ACR11 *is similar to that of *GLN2 *in various organs from 6-week-old *Arabidopsis*. Moreover, the expression of *ACR11 *and *GLN2 *is highly co-regulated by sucrose and light/dark treatments in 2-week-old *Arabidopsis *seedlings.

**Conclusions:**

This study reports the identification of four novel ACT domain repeat proteins, ACR9 to ACR12, in *Arabidopsis*. The ACR11 and ACR12 proteins are localized to the chloroplast, and the expression of *ACR11 *and *GLN2 *is highly coordinated. These results suggest that the *ACR11 *and *GLN2 *genes may belong to the same functional module. The *Arabidopsis *ACR11 protein may function as a regulatory protein that is related to glutamine metabolism or signaling in the chloroplast.

## Background

Nitrogen is one of the most important nutrients for plant growth and development. Plants can utilize different forms of nitrogen including nitrate, ammonium, and amino acids. Most plants use inorganic nitrogen nitrate as the primary nitrogen source. Nitrate taken up from the soil will be reduced to ammonium by nitrate reductase and nitrite reductase. Ammonium derived from nitrate or remobilized from the other nitrogen-containing compounds can be assimilated into glutamine and glutamate via the glutamine synthetase (GS)/glutamine-oxoglutarate aminotransferase (GOGAT) cycle. Glutamine and glutamate are the major amino donors for the synthesis of the other amino acids and nitrogen-containing compounds in plants [1]. In addition to their roles in protein synthesis and metabolism, glutamine and glutamate may also serve as signaling molecules in plants [2-6].

The synthesis of glutamine and glutamate also depends on the availability of α-ketoglutarate. In bacteria, the carbon skeleton of ammonia assimilation, α-ketoglutarate, signals nitrogen deficiency, whereas glutamine, the fully aminated product, often signals nitrogen sufficiency [7]. In *E. coli*, the expression of glutamine synthetase gene and its enzyme activity are regulated by the availability of glutamine and α-ketoglutarate [7-10]. In response to low glutamine/α-ketoglutarate, the *E. coli *PII protein (encoded by *glnB*) is uridylylated by GlnD, an uridylyltransferase/uridylyl-removing enzyme [11,12]. The uridylylated PII interacts with an adenylyltransferase to deadenylylate and activate the GS enzyme (encoded by *glnA*) [11,13]. In addition, the NtrB/NtrC two-component system will activate the expression of *glnA *under nitrogen-limiting conditions [9,14-19]. By contrast, in response to high glutamine/α-ketoglutarate, the uridylylated PII is deuridylylated by GlnD. The unmodified PII protein interacts with adenylyltransferase thereby causing the adenylylation and inactivation of the GS enzyme [11,12]. The unmodified PII protein also interacts with the NtrB/NtrC two-component system to inactivate the expression of *glnA *[9,14-19]. Thus bacterial PII proteins are sensors of α-ketoglutarate and adenylate energy charge, whereas GlnD is the sensor of glutamine [20,21].

Little is known about amino acid sensing and signaling in plants. PII-like proteins have been identified in *Arabidopsis *and rice [22,23]. However, bacterial GlnD homologs have yet to be identified in plants. The *E. coli *sensor protein GlnD is composed of a nucleotide transferase domain, a nucleotide hydrolase domain, and two C-terminal ACT domains. It has been shown that the C-terminal ACT domains of GlnD may regulate its activity through the binding of glutamine [21].

The ACT domain, named after bacterial aspartate kinase, chorismate mutase and TyrA (prephenate dehydrogenase), is a regulatory domain that serves as an amino acid-binding site in feedback-regulated amino acid metabolic enzymes [24-28]. For instance, the *E. coli *3-phosphoglycerate dehydrogenase (PGDH), a key enzyme in serine biosynthesis, is feedback regulated by serine. The C-terminal ACT domain of *E. coli *PGDH is the binding site for its allosteric effector serine [24,29,30]. The other amino acid metabolic enzymes such as acetohydroxyacid synthase [31], threonine deaminase [32,33], and phenylalanine hydroxylase [34] also contain the regulatory ACT domain. In addition, the ACT domain is also found in several transcription factors [35-39].

We previously identified a novel type of ACT domain-containing protein family in *Arabidopsis*, whose members contain four ACT domain repeats (the "ACR" protein family) [40]. Other than the ACT domain, the amino acid sequences of the ACR proteins do not have homology to any known enzymes or motifs in the database (http://www.ebi.ac.uk/Tools/InterProScan/). Although proteins homologous to the ACR family have been identified in rice [41-43], the functions of these ACR proteins are largely unknown.

In this report, we have identified four additional ACT domain-containing proteins in *Arabidopsis*. These proteins are composed of three or two copies of the ACT domain. The amino acid sequences of these proteins do not have any recognizable motifs except the ACT domain. These novel ACT domain-containing proteins are classified as new members of the ACR family. We showed that the newly identified ACR11 and ACR12 proteins are localized to the chloroplast. Interestingly, the expression of *ACR11 *is co-regulated with *GLN2 *that encodes a chloroplastic glutamine synthetase (GS). The possible functions of *Arabidopsis *ACR11 are discussed herein.

## Results

### Identification of four novel *ACR *genes in *Arabidopsis*

**Figure 1:**
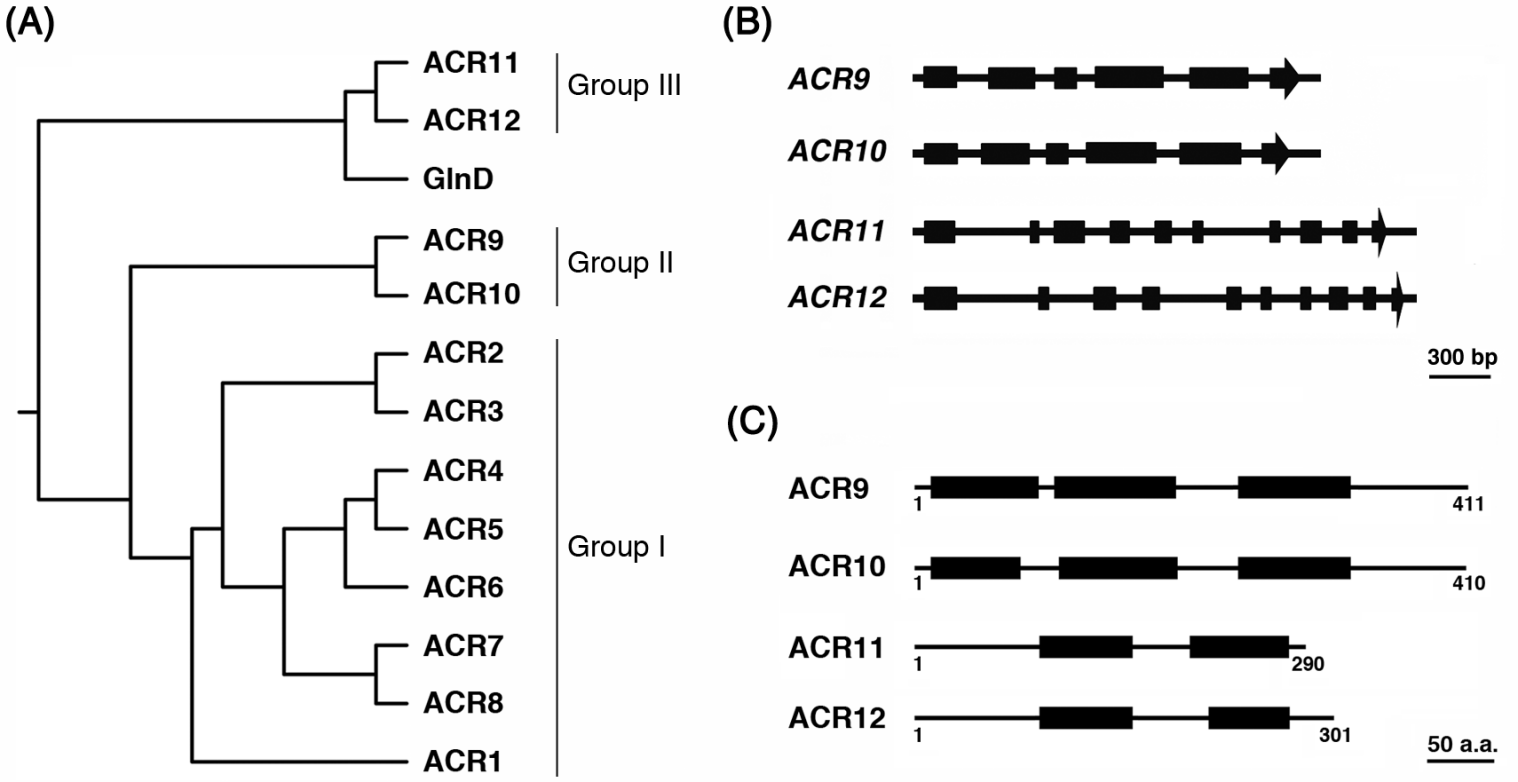
**Sequence analysis of the *Arabidopsis ACR *family**. **(A) **Phylogenetic relationships of *Arabidopsis *ACR proteins and the C-terminal ACT domains of *E. coli *GlnD. Full-length amino acid sequences of *Arabidopsis *ACR1 to ACR12 and amino acid residues 708-890 of *E. coli *GlnD were aligned by ClustalW2 and the neighbor-joining algorithm was used to obtain the phylogenetic tree. **(B) **Schematic gene structures of *Arabidopsis ACR9 *to *ACR12*. Exons are shown as black boxes and introns are indicated as solid lines. **(C) **Schematic diagram of *Arabidopsis *ACR9 to ACR12 proteins. The black boxes indicate the ACT domains.

We previously used the ACT domain (Pfam01842) and bacterial GlnD sequences to identify *Arabidopsis *ACR1 to ACR8 proteins, which contain four copies of the ACT domain [40]. In addition to these ACR proteins, we have identified four novel ACT domain-containing proteins encoded by *At1g16880*, *At2g36840*, *At2g39570 *and *At5g04740*, which contain two or three copies of the ACT domain. Since these proteins also contain ACT domain repeats, we propose to classify these proteins as new members of the ACR family. We named the proteins encoded by *At2g39570*, *At2g36840*, *At1g16880 *and *At5g04740 *genes ACR9, ACR10, ACR11 and ACR12, respectively. According to amino acid sequence alignment and phylogenetic analysis, ACR1 to ACR12 proteins are divided into three groups (Figure 1A). The originally identified ACR1 to ACR8 proteins belong to Group I. The newly identified ACR9 to ACR12 belong to Group II (ACR9 and ACR10) and Group III (ACR11 and ACR12), respectively (Figure 1A).

*ACR9 *and *ACR10 *have almost identical gene structures with respect to size and arrangement of their exons and introns (Figure 1B). By contrast, *ACR11 *and *ACR12 *have the same numbers of exon and intron, but some of the introns are different in size (Figure 1B). We used the computer program InterProScan (http://www.ebi.ac.uk/Tools/InterProScan/) to analyze domain compositions of ACR9 to ACR12. The ACR9 and ACR10 proteins contain three copies of the ACT domain, whereas the ACR11 and ACR12 proteins contain two copies of the ACT domain (Figure 1C). Similar to the ACR1 to ACR8 proteins, the ACR9 to ACR12 proteins do not have other known domains or motifs as revealed by InterProScan.

### Sequence analysis of *Arabidopsis *ACR11 and ACR12

**Figure 2:**
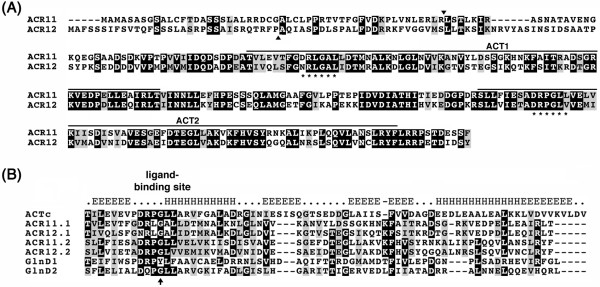
**Amino acid sequence alignments of ACR proteins and ACT domains**. **(A) **Sequence alignment of *Arabidopsis *ACR11 and ACR12 proteins. ACT domains are indicated with solid lines above the sequences. Arrowheads indicate the predicted cleavage sites of chloroplast transit peptides. Asterisks shown below the sequences denote the putative ligand-binding sites. **(B) **Sequence alignment of ACT consensus sequence (ACTc) from Pfam01842, and ACT domains from ACR11 (ACR11.1 and ACR11.2), ACR12 (ACR12.1 and ACR12.2) and GlnD (GlnD1 and GlnD2). The predicted secondary structure of the ACTc is shown above the sequences. Arrow indicates the conserved glycine residue in the β1-α1 loop region. Identical and similar amino acid residues are shaded in black and gray, respectively.

According to the sequences in the GenBank, we designed specific primers and used RT-PCR to amplify full-length cDNAs of *ACR11 *and *ACR12*. The ACR11 and ACR12 proteins have 290 and 301 amino acid residues, respectively. Amino acid sequence alignment of ACR11 and ACR12 shows that the N-terminal regions of these two proteins are not highly conserved. Beyond the N-terminal regions, the amino acid sequences in ACR11 (residues 74 to 290) and ACR12 (residues 85 to 301), share 63% identity and 82% similarity (Figure 2A). Several computer programs including PSORT (http://www.psort.org/) and TargetP (http://www.cbs.dtu.dk/services/TargetP/) predicted that the ACR11 and ACR12 proteins are localized to the chloroplast. Most nuclear-encoded chloroplast proteins contain N-terminal transit peptide sequences that facilitate the transfer of these proteins from the cytoplasm to the chloroplast. The transit peptides will be cleaved after the precursor proteins are imported into chloroplasts. In ACR11 and ACR12, the less conserved N-terminal sequences may function as transit peptides to target these proteins to the chloroplast. Indeed, the computer program ChloroP (http://www.cbs.dtu.dk/services/ChloroP/) predicts the presence of transit peptides in both proteins, and the locations of potential transit peptide cleavage sites are between the 52Arg-53Leu of ACR11, and the 32Pro-33Ala of ACR12, respectively (Figure 2A).

Protein BLAST analyses revealed that ACR11 and ACR12 are most similar to the ACT domains of bacterial PII-uridylyltransferase (GlnD) in addition to their homologs in photosynthetic organisms (data not shown). We aligned the ACT domains from *Arabidopsis *ACR11 and ACR12 with the two ACT domains from *E. coli *GlnD and the ACT consensus sequence from Pfam01842. The structure of the ACT consensus sequence is predicted to form a βαββαβ fold, which is in accordance with the archetypical structure of the ACT domain of *E. coli *PGDH [24]. In addition, the initial identification and alignment of ACT domains uncovered a nearly invariant Gly residue at the turn between the first β strand and the first α helix that coincided with the binding site for Ser in *E. coli *PGDH [25]. The alignment of ACT domains from ACR11, ACR12 and GlnD indicated that these sequences are highly conserved in the β1-α1 loop region (Figure 2B). Moreover, the invariant Gly residue is also present in the ACT domains of *Arabidopsis *ACR11 and ACR12 (Figure 2B).

### The ACR11- and ACR12-GFP are localized to the chloroplast

**Figure 3:**
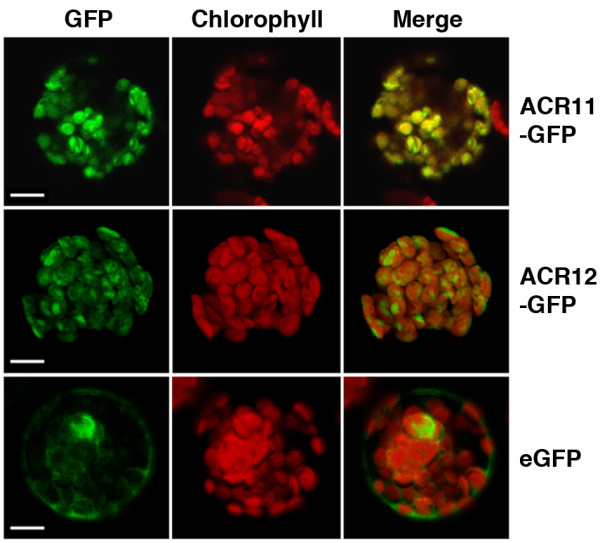
**The *Arabidopsis *ACR11- and ACR12-GFP fusion proteins are localized to the chloroplast**. *Arabidopsis *mesophyll protoplasts were transformed with ACR11- and ACR12-GFP constructs, which encode the full-length ACR11 protein, and the first 94 amino acids of ACR12 fused to GFP, respectively. Chloroplasts were visualized by red chlorophyll autofluorescence. The green fluorescent signals of ACR11- and ACR12-GFP colocalized with the red fluorescent signals of chlorophyll (merge). *Arabidopsis *protoplasts transformed with the empty vector are shown as controls for the subcellular localization of GFP. Scale bars are 10 μm.

We used green fluorescent fusion protein (GFP) and protoplast transient expression assay to examine the subcellular localization of ACR11 and ACR12. The full-length ACR11 and the first 94 amino acids of ACR12 were fused to the N-terminus of a GFP. The resulting ACR11- and ACR12-GFP fusion constructs driven by a cauliflower mosaic virus (CaMV) 35S promoter were transformed into *Arabidopsis *protoplasts. Confocal microscopy was used to observe the fluorescent signals 16 h after transformation. The green fluorescent signals of ACR11- and ACR12-GFP fusion proteins co-localized with the auto-fluorescent signals of chlorophylls in the chloroplasts (Figure 3). By contrast, the protoplast transformed with the empty GFP vector alone has green fluorescent signals in the cytosol and nucleus (Figure 3). These results suggest that the *Arabidopsis *ACR11 and ACR12 proteins are localized to the chloroplast.

### Coexpression gene networks of *Arabidopsis ACR11 *and *ACR12*

**Figure 4:**
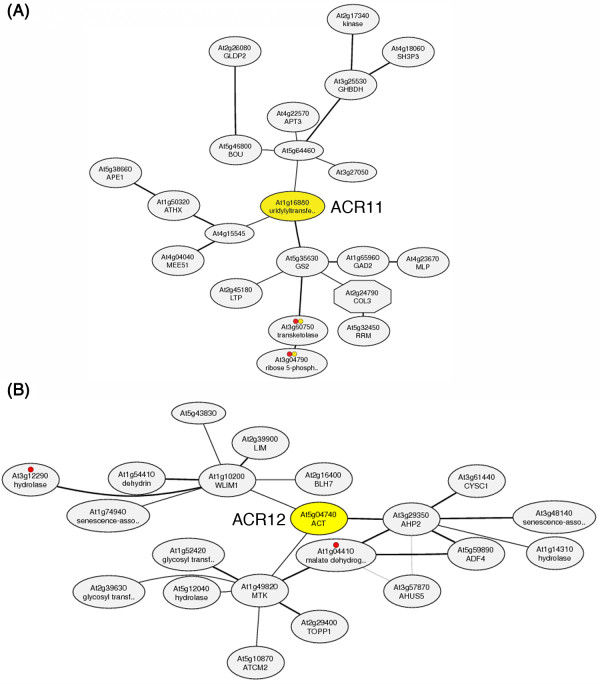
**Coexpressed gene networks around *Arabidopsis ACR11 *and *ACR12***. **(A) **The three genes having the highest coexpression mutual rank (MR) with *ACR11 *are *At5g35630 *(encoding glutamine synthetase 2; MR = 1.0), *At4g15545 *(encoding an unknown protein; MR = 8.5) and *At5g64460 *(encoding an unknown protein; MR = 9.2). The ACR11 (At1g16880) is annotated as an uridylyltransferase-related protein in the database. **(B) **The three genes having the highest coexpression mutual rank (MR) with *ACR12 *are *At3g29350 *(encodes AHP2, histidine-containing phosphotransmitter2; MR = 2.2), *At1g10200 *(encodes WLIM1, a member of the *Arabidopsis *LIM proteins; MR = 6.2), and *At1g49820 *(encodes MTK1, 5-methylthioribose kinase1; MR = 7.5). The coexpression gene networks of *ACR11 *and *ACR12 *can be obtained at the ATTED-II website (http://atted.jp/data/locus/At1g16880.shtml and http://atted.jp/data/locus/At5g04740.shtml).

The functions of *Arabidopsis *ACR11 and ACR12 are completely unknown. It has been suggested that genes involved in related biological pathways are often expressed cooperatively [44]. We attempted to identify the functions of ACR11 and ACR12 by searching for genes that are coexpressed with *ACR11 *and *ACR12*, respectively. We obtained the *ACR11 *and *ACR12 *coexpression gene networks from the ATTED-II database (http://atted.jp/) [45]. The three genes having the highest mutual rank (MR) with *ACR11 *are *At5g35630 *(*GLN2*, encodes a chloroplastic glutamine synthetase; MR = 1.0), *At1g15545 *(encodes an unknown protein; MR = 8.5), and *At5g64460 *(encodes an unknown protein; MR = 9.2) (Figure 4A). It is intriguing to find that *ACR11 *and *GLN2 *have the highest mutual rank of coexpression compared with all other genes in the *Arabidopsis *genome. By contrast, the top three genes that are coexpressed with *ACR12 *are *At3g29350 *(encodes AHP2, histidine-containing phosphotransmitter2; MR = 2.2), *At1g10200 *(encodes WLIM1, a member of the *Arabidopsis *LIM proteins; MR = 6.2), and *At1g49820 *(encodes MTK1, 5-methylthioribose kinase1; MR = 7.5) (Figure 4B). The expression of *ACR12 *is not co-ordinately regulated with *ACR11 *and *GLN2 *in the ATTED-II database.

### The expression of *ACR11 *and *GLN2 *is up-regulated by light and sucrose

**Figure 5:**
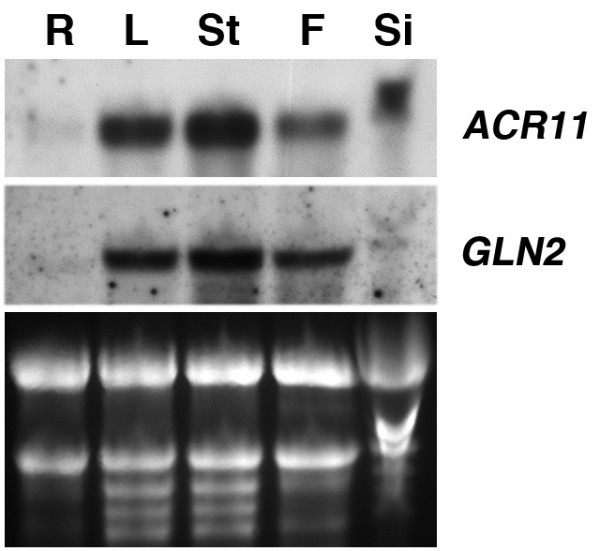
**Expression patterns of *ACR11 *and *GLN2 *in *Arabidopsis***. Total RNA (10 μg) from roots (R), leaves (L), stems (St), flowers (F), and siliques (Si) of 6-week-old *Arabidopsis *grown in soils was used for RNA gel blot analysis. The ethidium bromide-stained agarose gel of the same samples is shown at the bottom.

**Figure 6:**
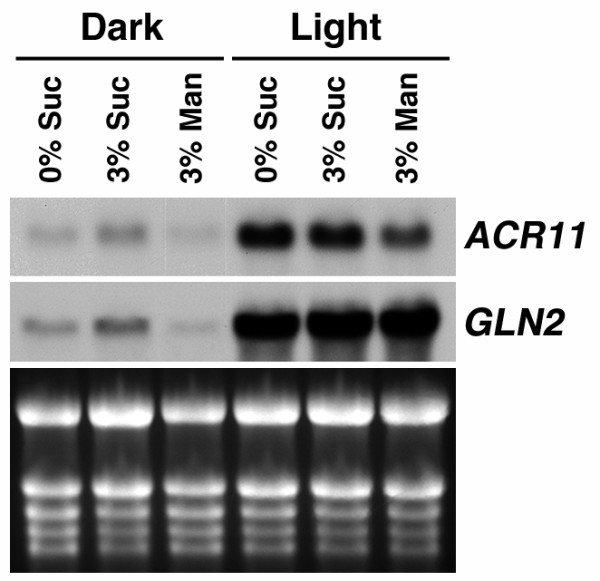
**The expression of *Arabidopsis ACR11 *and *GLN2 *is co-regulated by sucrose and light/dark treatments**. Total RNA (10 μg) from 14-day-old *Arabidopsis *plants treated with complete darkness or continuous light for 48 h was used for RNA gel blot analysis. During the dark or light treatment, plants were grown on MS media containing 0% sucrose, 3% sucrose, or 3% mannitol. The expression of *ACR11 *and *GLN2 *is up-regulated by sucrose and light.

We used RNA gel blot analysis to examine the expression patterns of *ACR11 *and *GLN2 *in different organs from 6-week-old *Arabidopsis *plants. Steady-state levels of *ACR11 *and *GLN2 *mRNAs are low in roots compared to those of leaves, stems, and flowers (Figure 5). It is well known that the expression of *Arabidopsis GLN2 *is regulated by light and sucrose [46]. We used RNA gel blot analysis to examine the effects of light and sucrose on the expression of *ACR11 *and *GLN2 *(Figure 6). Two weeks old *Arabidopsis *seedlings grown on a 16 h light/8 h dark cycle were transferred to media containing 0% sucrose, 3% sucrose or 3% manitol, and dark-adapted or grown in continuous light for 48 h. Total RNA extracted from these samples was used for RNA gel blot analysis. In dark-adapted seedlings, steady-state levels of *ACR11 *and *GLN2 *mRNAs are slightly increased by 3% sucrose treatment. This sucrose effect is not related to an osmotic change, because the addition of 3% mannitol does not increase the accumulation of *ACR11 *and *GLN2 *transcripts. By contrast, steady-state levels of *ACR11 *and *GLN2 *mRNAs are significantly increased by the light treatment, regardless of the amounts of sucrose or mannitol in the media. The expression patterns of *ACR11 *and *GLN2 *are almost identical under these treatments. These results confirm that the *ACR11 *and *GLN2 *genes are expressed cooperatively under various conditions.

### *ACR11 *promoter-GUS activity

**Figure 7:**
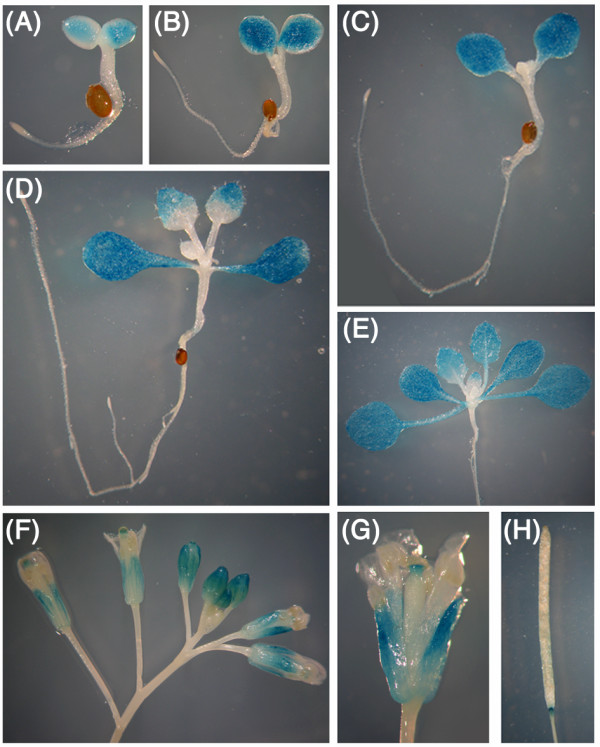
**GUS activity in transgenic *Arabidopsis *containing *ACR11 *promoter-GUS fusion**. **(A) **3-day-old, **(B) **5-day-old, **(C) **7-day-old, **(D) **10-day-old, **(E) **14-day-old seedlings. **(F) **Flower buds and mature flowers. **(G) **Close-up of a mature flower. **(H) **A mature silique.

To further examine the cell type and tissue specific expression of the *ACR11 *gene, we fused the putative promoter of *ACR11 *to a β-glucuronidase reporter gene (ACR11p-GUS) and generated stable *Arabidopsis *transgenic lines. The ACR11p-GUS activity was detected in the cotyledons of 3-, 5- and 7-day-old seedlings (Figure 7A-C). Interestingly, the ACR11p-GUS activity was not detected in emerging young leaves and the basal part of maturing leaves, which are mainly composed of dividing and growing young cells (Figure 7C-E). In developing or mature flowers, the ACR11p-GUS activity was detected in sepals as a gradient from the apical part (high) to the basal part (low) (Figure 7F and 7G). In mature flowers, the ACR11p-GUS activity was also detected in the style (Figure 7G). In mature siliques, the ACR11p-GUS activity was detected in the tip of the pedicel (Figure 7H).

## Discussion

### Three distinct groups of ACR proteins in *Arabidopsis*

We previously reported the identification and characterization of eight ACT domain repeat proteins in *Arabidopsis *and named these proteins ACR1 to ACR8, respectively [40]. These ACR proteins each contain four copies of the ACT domain. Here, we describe four additional ACT domain-containing proteins in *Arabidopsis*. Except in the regions of the ACT domain, the amino acid sequences of these novel ACT domain-containing proteins are not similar to the originally identified ACR proteins. However, they also contain multiple copies of the ACT domain. We thus adopted the term "ACT domain repeats (ACR)" and named these proteins ACR9 to ACR12, respectively.

Amino acid sequence alignment and phylogenetic analysis clearly divided these ACR proteins into three different groups. The originally identified ACR1 to ACR8 proteins contain four copies of the ACT domain and belong to Group I. The ACR9 and ACR10 proteins have three copies of the ACT domain, which are classified as Group II ACR proteins. The amino acid sequences of ACR9 and ACR10 are very similar throughout the entire polypeptides. Moreover, the gene structures of *ACR9 *and *ACR10 *are almost identical (Figure 1B), which suggests that these two genes are recently duplicated in the *Arabidopsis *genome during evolution. By contrast, Group III ACR proteins, including ACR11 and ACR12, contain two copies of the ACT domain. The gene structures of *ACR11 *and *ACR12 *are similar. However, the encoded amino acid sequences are not conserved in the N-terminal regions. The rest of the amino acid sequences, e.g. residues 74 to 290 of ACR11, and residues 85 to 301 of ACR12, are highly conserved. The non-conserved N-terminal amino acid sequences of ACR11 and ACR12 are predicted to be transit peptides, which target these proteins to the chloroplast. Thus Group III ACR proteins may be localized to the chloroplast.

### Group III ACR proteins are localized to the chloroplast

Most amino acids are synthesized in the chloroplast. It is expected that some regulatory proteins involved in amino acid metabolism or signaling may also exist in the chloroplast. The *Arabidopsis *Group III ACR proteins are good candidates in this regard, because they are predicted to target to the chloroplast. We used transient expression assay in *Arabidopsis *protoplasts to verify that the ACR11- and ACR12-GFP fusion proteins are localized to the chloroplast (Figure 3). After the removal of transit peptide, the mature ACR11 and ACR12 proteins are only composed of two ACT domains. It is conceivable that the ACT domains of the ACR11 and ACR12 proteins may serve as amino acid binding domains. Upon binding to specific amino acids, the ACR11 and ACR12 proteins may regulate the activities of amino acid biosynthetic enzymes in the chloroplast. Alternatively, the two ACT domains of the ACR11 and ACR12 proteins may function as specific amino acid sensors in the chloroplast, which are similar to those of bacterial GlnD proteins. It will be interesting to further characterize the functions of the *Arabidopsis *ACR11 and ACR12 proteins and their homologs in the other plants.

### *ACR11 *and *GLN2 *are in the same coexpressed gene network

Genes involved in related biological pathways are often coordinately regulated [44]. The coexpression analysis obtained from the ATTED-II database (http://atted.jp) may help us to identify the functions of *Arabidopsis *ACR11 and ACR12. In the ATTED-II database, the *ACR11 *and *ACR12 *genes have distinct coexpressed gene networks (Figure 4). It is possible that the proteins encoded by these two homologous genes may also have distinct functions in *Arabidopsis *chloroplasts. It is intriguing that the *Arabidopsis ACR11 *and *GLN2 *are in the same coexpressed gene network. Moreover, the mutual rank for coexpression of these two genes is the highest in their respective gene networks (Figure 4). It is well known that the expression of *Arabidopsis GLN2 *is regulated by light and sugars [46]. We used RNA gel blot analysis to examine the effects of light and sucrose on the expression of *ACR11*. Interestingly, the results are in accordance with the coexpression analysis in the database. Steady-state levels of both *ACR11 *and *GLN2 *mRNAs are increased by treatments of sucrose and light (Figure 6). The highly cooperative expression of *ACR11 *and *GLN2 *observed in our experiments and in the database suggests that these two genes may belong to the same functional module. The *GLN2 *encodes a chloroplastic GS2, which is the major enzyme for glutamine synthesis in the chloroplast. However, the functions of the chloroplast-localized ACR11 protein are completely unknown. The *ACR11 *and *GLN2 *genes have the highest coexpression relationship in the *Arabidopsis *genome suggests that the ACR11 protein may have functions related to GS2.

The relationship between *Arabidopsis *ACR11 and GS2 is reminiscent of the PII-GlnD system in the regulation of *glnA *gene expression and GS enzyme activity in bacteria [7-10,18]. In addition to the ACR homologs in plants, the amino acid sequence of ACR11 is most similar to the ACT domains of the bacterial sensor protein GlnD (e.g. uridylyltransferase). Thus the ACR11 (At1g16880) was annotated as uridylyltransferase-related protein in the GenBank (NM_101549). The bacterial GlnD protein may sense the availability of glutamine, possibly via the two ACT domains in the C-terminal region, to regulate GS enzyme activity and its gene expression [21]. It is possible that the *Arabidopsis *ACR11 protein may also use its ACT domains to sense the availability of glutamine in the chloroplast, and then regulates GS2 activity or glutamine metabolism.

### ACR11 and ACR12, putative amino acid sensor proteins in the chloroplast

Chloroplast is the site of active primary and secondary nitrogen assimilation inside a plant cell. The assimilation of ammonia into glutamine is the major pathway to convert inorganic nitrogen into organic nitrogen in plants. Thus it is expected that plants may have a mechanism to sense the availability of glutamine inside the chloroplast. In *E. coli*, glutamine may serve as a signaling molecule to affect the expression of nitrogen assimilatory genes and the activities of nitrogen metabolic enzymes [7]. The two ACT domains located in the C-terminal region of the GlnD protein are considered as glutamine sensors in bacteria [21]. Little is known about amino acid sensing and signaling in plants. Interestingly, the ACR11 and ACR12 proteins are composed of two ACT domains, and are localized to the chloroplast. It is conceivable that the ACR11 and ACR12 proteins may function as amino acid sensors in *Arabidopsis*. Future studies are needed to determine the functions of these chloroplastic ACR proteins.

## Conclusions

Although the ACT domains have high sequence divergence, there is a common regulatory theme among these domains. The *Arabidopsis *ACR proteins contain multiple copies of the ACT domain and their functions are largely unknown. In this study, we identified two new groups of ACR proteins in *Arabidopsis*. Group II ACR proteins, ACR9 and ACR10, have three copies of the ACT domain. Whereas group III ACR proteins, ACR11 and ACR12, contain two copies of the ACT domain, and are localized to the chloroplast. The activities of *ACR11 *promoter-GUS are mainly detected in mature leaves. Moreover, the expression of *ACR11 *and *GLN2 *is highly coordinated. The ACR11 may function as a regulatory protein involved in glutamine metabolism or sensing in *Arabidopsis*.

## Methods

### Plant material and growth conditions

*Arabidopsis thaliana *ecotype Columbia-0 was grown in soils in the greenhouse on a 16-h light/8-h dark cycle at 23°C. Roots, leaves, stems, flowers, and siliques from the same batch of 6-week-old soil-grown plants were used for total RNA extraction. For experiments in which plants were transferred to 0%, 3% sucrose or 3% mannitol, seeds were sown on 1.5 cm × 8 cm Nylon nets with 250 μm mesh size (Tetko, Elmsford, NY, USA, catalog no. 3-250/50), placed on the surface of the Murashige and Skoog (MS) plates [MS salts (Sigma-Aldrich Co., St. Louis, MO), pH adjusted to 5.7 with 1N KOH, 0.8% (w/v) phytoagar] containing 3% sucrose. After cold treatment at 4°C for 48 h, plates were vertically placed in a 23°C chamber on a 16-h light/8-h dark cycle for two weeks. The plants and the nylon nets were lifted and transferred to fresh MS media containing 0%, 3% sucrose or 3% mannitol, and dark-adapted or grown in continuous light for 48 h.

### Cloning of Arabidopsis *ACR9*, *ACR10*, *ACR11 *and *ACR12 *cDNAs

Total RNA from 2-week-old *Arabidopsis *was used for reverse transcription-PCR (SuperScript II RT Kit, Invitrogen, Carlsbad, CA) to amplify *ACR9 *(*At2g39570*), *ACR10 *(*At2g36840*), *ACR11 *(*At1g16880*) and *ACR12 *(*At5g04740*) cDNAs. The following primers were used to amplify full-length cDNAs: *ACR9*, 5'-TGTTGTTGATTCATTGGCTC-3' and 5'-AGTAGTAGATGAATATATTG-3'; *ACR10*, 5'-ATAGGAGGAACAACACAAAC-3' and 5'-TTACTATGAAACCCACACAG-3'; *ACR11*, 5'-AAAAGGATCCATGGCTATGGCCTCTGCTTC-3' and 5'-GGGGAGGCCTGAAACTTGACTCGTCAGTTG-3'; *ACR12*, 5'-AGGGACCGGTATGGCGTTCTCGAGTTCCAT-3' and 5'-GGGGACCGGTGTAGCTGTCAATGTCAGTTT-3'. The PCR products were cloned into pGEM-T easy vector (Promega Co., Madison, WI) and provided for sequencing. The *Arabidopsis ACR9 *to *ACR12 *cDNA sequences were verified and deposited in the GenBank (JF797174 to JF797177).

### Sequence analysis

The amino acid sequences of *Arabidopsis *ACR1 (NM_125986), ACR2 (NM_122441), ACR3 (NM_179566), ACR4 (NM_202378), ACR5 (NM_126420), ACR6 (NM_111065), ACR7 (NM_118407), ACR8 (NM_101114), ACR9 (JF797174), ACR10 (JF797175), ACR11 (JF797176), ACR12 (JF797177), and amino acid residues 708-890 of *E. coli *GlnD (M96431) were aligned by ClustalW2 with default settings (http://www.ebi.ac.uk/Tools/msa/clustalw2/). The neighbor-joining algorithm was used to obtain the phylogenetic tree. The sequence alignment was shaded with BOXSHADE 3.21 (http://www.ch.embnet.org/software/BOX_form.html). InterProScan (http://www.ebi.ac.uk/Tools/pfa/iprscan/) was used to analyze the domain composition of ACR9 to ACR12. PSORT (http://www.psort.org/) and TargetP (http://www.cbs.dtu.dk/services/TargetP/) were used to predict the subcellular localization of ACR9 to ACR12. ChloroP (http://www.cbs.dtu.dk/services/ChloroP/) was used to predict the transit peptide cleavage sites of ACR11 and ACR12. The *ACR11 *and *ACR12 *coexpression gene networks were obtained from the ATTED-II database (http://atted.jp/).

### ACR11- and ACR12-GFP fusion constructs

The GFP expression vector pHBT, designed for transient expression assays [47], was used to construct the ACR11- and ACR12-GFP fusions. A *Bam*HI/*Stu*I fragment from the pGEM-T-*ACR11 *clone containing the full-length *ACR11 *cDNA was subcloned into the pHBT vector to create an ACR11-GFP fusion construct. The N-terminal cDNA sequence encoding the first 94 amino acids of ACR12 was amplified by PCR using primers 5'-GGAAGGATCCATGGCGTTCTCGAGTTCCATC-3' and 5'-GGAAAGGCCTCATTGGAACAACGTCGTCATC-3'. The PCR product was digested with *Bam*HI and *Stu*I, and cloned into the N-terminus of the GFP in the pHBT vector. The resulting construct, ACR12-GFP, contains the putative transit peptide of ACR12 fused to a GFP. The obtained ACR11- and ACR12-GFP constructs, and the GFP empty vector were transformed into *Arabidopsis *protoplasts using polyethylene glycol (PEG)-mediated transient gene expression [47] and observed under confocal laser scanning microscope (510 META Zeiss) 16 h after transformation.

### RNA gel blot analysis

*Arabidopsis *total RNA was isolated using a phenol extraction protocol [48]. Total RNA (10 μg) was separated in standard formaldehyde gel by electrophoresis and blotted onto a nylon membrane. For detection of *ACR11 *and *GLN2 *mRNA, digoxigenin (DIG)-labeled single-stranded DNA probes were generated by PCR using the following primers: *ACR11 *(*At1g16880*), 5'-ATGGCTATGGCCTCTGCTTC-3', 5'-GAAACTTGACTCGTCAGTTG-3'; *GLN2 *(*At5g35630*), 5'-GGTGAAGTTATGCCTGGA-3', 5'-GAGAGACCACATAGACAC-3'. DIG probe labeling, pre-hybridization, hybridization, wash conditions and detection were performed according to the Boehringer-Mannheim Genius System User's Guide: DIG Application Manual for Filter Hybridization.

### *ACR11 *promoter-GUS fusion

*ACR11 *(*At1g16880*) and its upstream gene *At1g16870 *are in an opposite orientation. There are 638 nucleotides between the initiation codons (ATG) of these two genes. The putative promoter of *ACR11 *(-1 to -625 of the start codon) was amplified from the *Arabidopsis *genomic DNA by PCR using the primers 5'-CACCTCTAGACACTCAAAAATCGGAATTAA-3' and 5'-AACAAAGCTTATCTCTTGAGTCTGACTCAA-3'. The PCR product was cloned into the pCR2.1-TOPO vector (TOPO TA Cloning Kit, Invitrogen) and the sequence was confirmed. A *Hin*dIII/*Xba*I fragment containing the 0.625 kb *ACR11 *promoter region was subcloned into the pBI101 binary vector to create an *ACR11 *promoter-GUS fusion construct that was transformed into the *Agrobacterium tumefaciens *strain GV3101.

The floral dip method was used for *Arabidopsis *transformation [49]. Several independent *ACR11 *promoter-GUS *Arabidopsis *transgenic lines were grown to T3 homozygous and stained for GUS activity [50].

## Authors' contributions

TYS carried out protoplast transient assays. TYC carried out RNA blot analysis. TYS, TYC and CPH participated in molecular cloning and promoter-GUS analysis. MHH conceived the study, carried out bioinformatic analysis and sequence alignment, and wrote the manuscript. All authors read and approved the final manuscript.
